# Automated calibration and control for polarization-resolved second harmonic generation on commercial microscopes

**DOI:** 10.1371/journal.pone.0195027

**Published:** 2018-04-10

**Authors:** Elisabeth I. Romijn, Andreas Finnøy, Rajesh Kumar, Magnus B. Lilledahl

**Affiliations:** Department of Physics, Norwegian University of Science and Technology, Trondheim, Norway; Pennsylvania State Hershey College of Medicine, UNITED STATES

## Abstract

Polarization-resolved second harmonic generation (P-SHG) microscopy has evolved as a promising technique to reveal subresolution information about the structure and orientation of ordered biological macromolecules. To extend the adoption of the technique, it should be easily integrated onto commercial laser scanning microscopes. Furthermore, procedures for easy calibration and assessment of measurement accuracy are essential, and measurements should be fully automated to allow for analysis of large quantities of samples. In this paper we present a setup for P-SHG which is readily incorporated on commercial multiphoton microscopes. The entire system is completely automated which allows for rapid calibration through the freely available software and for automated imaging for different polarization measurements, including linear and circular polarization of the excitation beam. The results show that calibration settings are highly system dependent. We also show that the accuracy of the polarization control is easily quantified and that it varies between systems. The accuracy can be tuned by iterative alignment of optics or a more fine-grained calibration procedure. Images of real samples show that the red accuracy of the results is easily visualized with the automated setup. Through this system we believe that P-SHG could develop a wider adoption in biomedical applications.

## Introduction

Polarization-resolved second harmonic generation (P-SHG) is based on the dependency of the second harmonic generation (SHG) on the polarization of the excitation beam. The relation between the incoming electric field and the generated second harmonic field is governed by the second order susceptibility tensor. This tensor has molecular specificity, and depends on the orientation of the molecule compared to the incoming electric field. P-SHG extracts this information by acquiring the SHG signal as a function of the polarization angle of a linearly polarized excitation beam, and comparing the angular dependency of the intensity to a theoretical model [1]. P-SHG can be employed to distinguish between collagen types [2] and provide structural information, such as in-plane molecular orientation [3] and helical angles [4, 5], all at pixel level resolution [6]. This technique can be used to study any tissue containing an SHG source, and has previously been utilized to examine atherosclerotic lesions [7], liver fibrosis [8], breast tissue [9], tissue engineered cartilage [10] and cornea [11].

To enable P-SHG one has to include optical elements in the beam path that control the polarization state of the laser at the imaging plane. On a custom-built microscope this can be implemented by placing for example a polarizer or a quarter-wave plate (QWP), and half-wave plate (HWP) just before the objective [12]. Implementing a polarization control on a commercial microscope is more complicated. There is no room to add additional optics inside the microscope body, and any optical component in the microscope prior to the imaging plane, especially the dichroic mirror, modify the polarization state of the laser [13]. The polarization control has to compensate for the modification. The most commonly used setup is a combination of a polarizer, QWP and HWP [2, 14, 15]. Another approach is to rotate the sample stage instead of the polarization [14, 16]. However, a disadvantage of the later is that the P-SHG analysis is more complicated because the sample typically moves from frame to frame.

Manual calibration and adjustment of the polarization control on a commercial microscope is a tedious procedure, since both the QWP and HWP have to be calibrated and adjusted for every desired polarization state at the imaging plane [2]. In an attempt to simplify this process Brideau and Stys [17] implemented motorized control of the QWP and HWP. However, the software they designed still required the user to control the motors and manually search for the QWP and HWP combination that provides each desired polarization state at the imaging plane. Ideally the calibration process should be fully automated, where after the user can extract the required QWP and HWP combination for any polarization state. Furthermore, it must be easy to assess the calibration and the accuracy of the polarization control. Recent work in P-SHG attempts to detect minute variations in polarization signal [2, 10] and quantitative knowledge about the accuracy of the calibration is therefore essential.

The purpose of this study is to present a fully automated system for P-SHG analysis designed for commercial microscopes, with easy methods for calibration and assessment of polarization accuracy. We present the equipment, software, and theory required to implement and calibrate a polarization control on a commercial microscope. The software designed to calibrate the polarization control and automated P-SHG analysis is made freely available [18]. An analysis of the results and accuracy have been included to demonstrate the possibilities of an automated design. This includes assessment of the achieved linearity/circularity of the polarization by the calibration, evaluation of the accuracy of the provided QWP and HWP combinations and if it is possible to further improve the linearity/circularity, as well as inspection of tissue response to identical polarization states provided by different HWP and QWP settings.

## Theory

The polarization of the electric field in the image plane is controlled by a QWP and HWP placed prior to the beam entering the confocal scanner. However, optical components in the microscope prior to the imaging plane will further modify the polarization and may cause a relative change in both phase and attenuation between the orthogonal components of the electric field. The calibration procedure must compensate for these effects.

**Fig 1:**
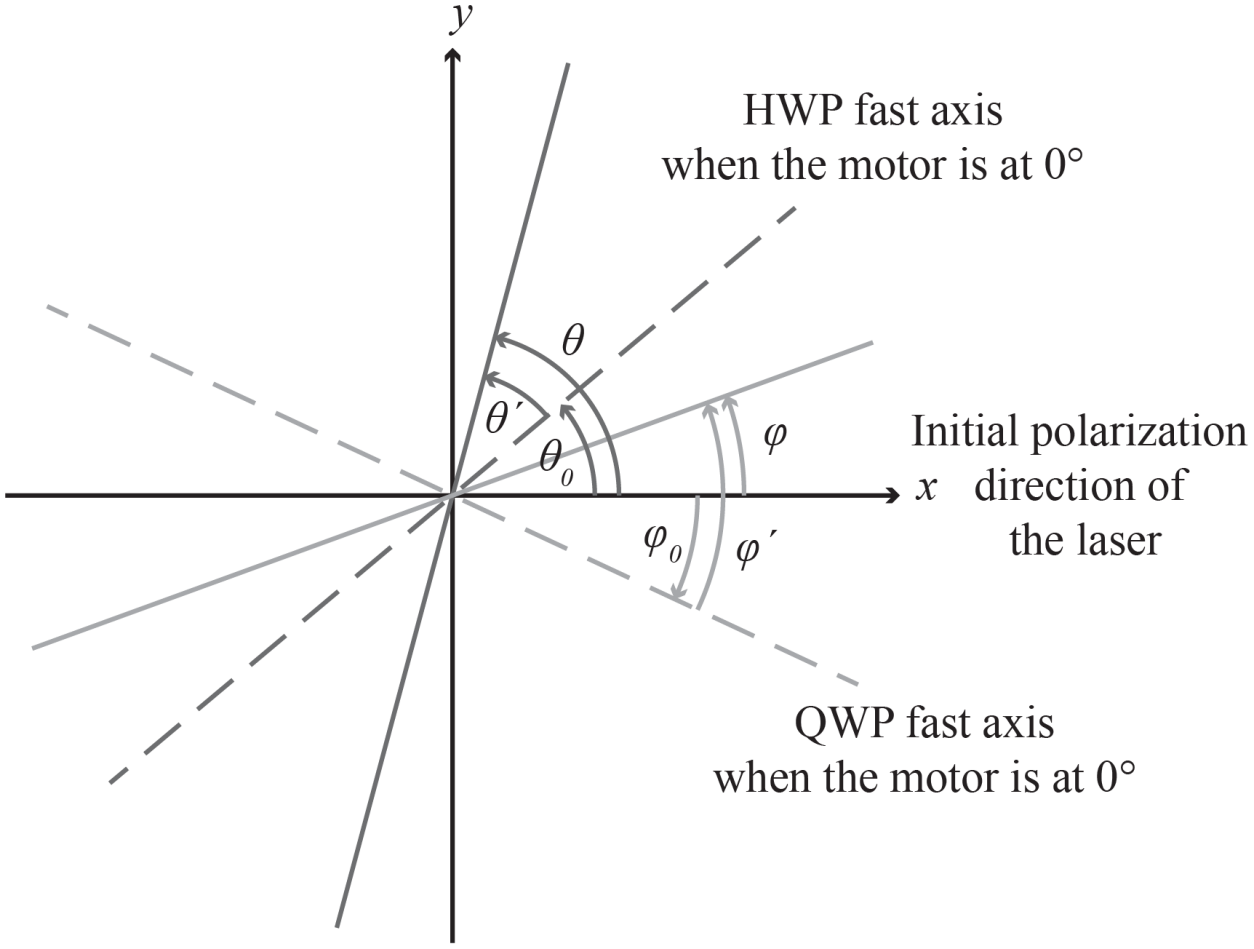
An illustration of the orientation and rotation of the QWP and HWP compared to the polarization of the incoming laser light. The motors containing the QWP and HWP are not calibrated according to the *x*-axis. Therefore, a relative angle *ϕ* = *ϕ*′ − *ϕ*_0_ and *θ* = *θ*′ − *θ*_0_ have to be implemented for the QWP and HWP, respectively. Here *ϕ*′ and *θ*′ are the angles provided by the motors, and *ϕ*_0_ and *θ*_0_ are the angles of the fast axis of the QWP and HWP, respectively, compared to the *x*-axis when the motors are at 0°.

Let the *x*-axis be defined by the polarization of the incoming light before passing the waveplates (refer to Fig 1). Further, assume that the incoming light (with frequency *ω*), first passes through a QWP followed by a HWP, with the fast axis oriented at an angle of *ϕ* and *θ*, respectively, relative to the *x*-axis, and that the optical components in the microscope introduce a relative change in phase, *δ*, and attenuation, *γ*, between the *x* and *y* components of the electric field. Based on this model the electric field at the imaging plane can then be expressed as

$$\begin{array}{ll} \vec{E} & =E_{0}e^{i\omega t}\left\{E_{x}\hat{x}+E_{y}\hat{y}\right\}\\ & =E_{0}e^{i\omega t}\left\{\left[d_{1}+id_{2}\right]\hat{x}+\left[d_{3}+id_{4}\right]\hat{y}\right\}, \end{array}$$ (1)

where

$$\begin{array}{ll} d_{1} & =-\gamma \left[\text{cos}\delta \text{sin}\phi \text{sin}\left(2\theta -\phi \right)+\text{sin}\delta \text{cos}\phi \text{cos}\left(2\theta -\phi \right)\right]\\ d_{2} & =-\gamma \left[\text{sin}\delta \text{sin}\phi \text{sin}\left(2\theta -\phi \right)-\text{cos}\delta \text{cos}\phi \text{cos}\left(2\theta -\phi \right)\right]\\ d_{3} & =\text{sin}\phi \text{cos}\left(2\theta -\phi \right)\\ d_{4} & =\text{cos}\phi \text{sin}\left(2\theta -\phi \right). \end{array}$$ (2)

The derivation follows along the same lines as Chou et al. [15] (we are assuming a plane wave, i.e. in the absence of an objective).

However, it is impractical to have to calibrate the motorized stages that hold the QWP and HWP such that the fast axis of the waveplates are initially aligned with the *x*-axis. The angles of the waveplates are therefore replaced by *ϕ* = *ϕ*′ − *ϕ*_0_ and *θ* = *θ*′ − *θ*_0_, where *ϕ*′ and *θ*′ are the angles provided by the motors, and *ϕ*_0_ and *θ*_0_ is the angular difference between the fast axis and the *x*-axis at the 0° position of the motors (see Fig 1).

At the imaging plane an analyzer combined with a power meter measure the intensity at different angles *α* of the analyzer

$$\begin{array}{c} I_{\alpha }=E_{x}E_{x}^{*}\cos^{2}\alpha +E_{y}E_{y}^{*}\sin^{2}\alpha +(E_{x}E_{y}^{*}+E_{x}^{*}E_{y})\sin\alpha \cos\alpha \;. \end{array}$$ (3)

Here *E*_*x*_ and *E*_*y*_ refers to the components of the electric field described in Eq 1. Similar to the HWP and QWP angles, it is not convenient to calibrate the motorized stage holding the analyzer to the *x*-axis. Therefore the angle of the analyzer is expressed as *α* = *α*′ − *α*_0_, where *α*′ is the angles provided by the motor, and *α*_0_ is the relative angle between the transmitting axis of the analyzer and the *x*-axis at the 0° position of the motor.

As explained by Chou et. al. [15] the electric field at the imaging plane is linearly polarized if $\frac{d_{3}}{d_{1}}=\frac{d_{4}}{d_{2}}$, resulting in the condition

$$\begin{array}{c} \tan(2\phi )=-\tan\delta \sin(2(2\theta -\phi ))\;. \end{array}$$ (4)

Derived in similar manner, the electric field is circular if *d*_3_ = ∓*d*_2_ and *d*_4_ = ±*d*_1_, providing the relations

$$\begin{array}{c} \tan\left(2\theta -\phi \right)=\frac{\gamma \cos\delta \pm \tan\phi }{\gamma \sin\delta \tan\phi } \end{array}$$ (5)

and

$$\begin{array}{c} \tan\left(2\theta -\phi \right)=\frac{-\gamma \sin\delta }{\gamma \cos\delta \tan\phi \pm 1}\;, \end{array}$$ (6)

which both have to be fulfilled for the polarization to be circular. The signs in Eqs 5 and 6 are related and should therefore be paired as indicated. Changing the signs will change the handedness of the circularly polarized light.

The polarization dependence at the imaging plane can also be described in terms of the maximum and minimum intensities as opposed to the field components along certain axes as in Eq 3.

$$\begin{array}{c} I_{\alpha }=E_{max}^{2}\cos^{2}\left(\alpha_{max}-\alpha \right)+E_{min}^{2}\sin^{2}\left(\alpha_{max}-\alpha \right)\;. \end{array}$$ (7)

Here *α* is the analyzer angle relative to the *x*-axis and *E*_*max*_, which is found at an angle of *α*_*max*_, and *E*_*min*_ are the maximum and minimum electric field amplitudes at the imaging plane. The maximum and minimum field intensities are determined by fitting the polarization measurements to the above equation to yield the degree of polarization *ρ* = *E*_*min*_/*E*_*max*_.

## Materials and methods

### Equipment

**Fig 2:**
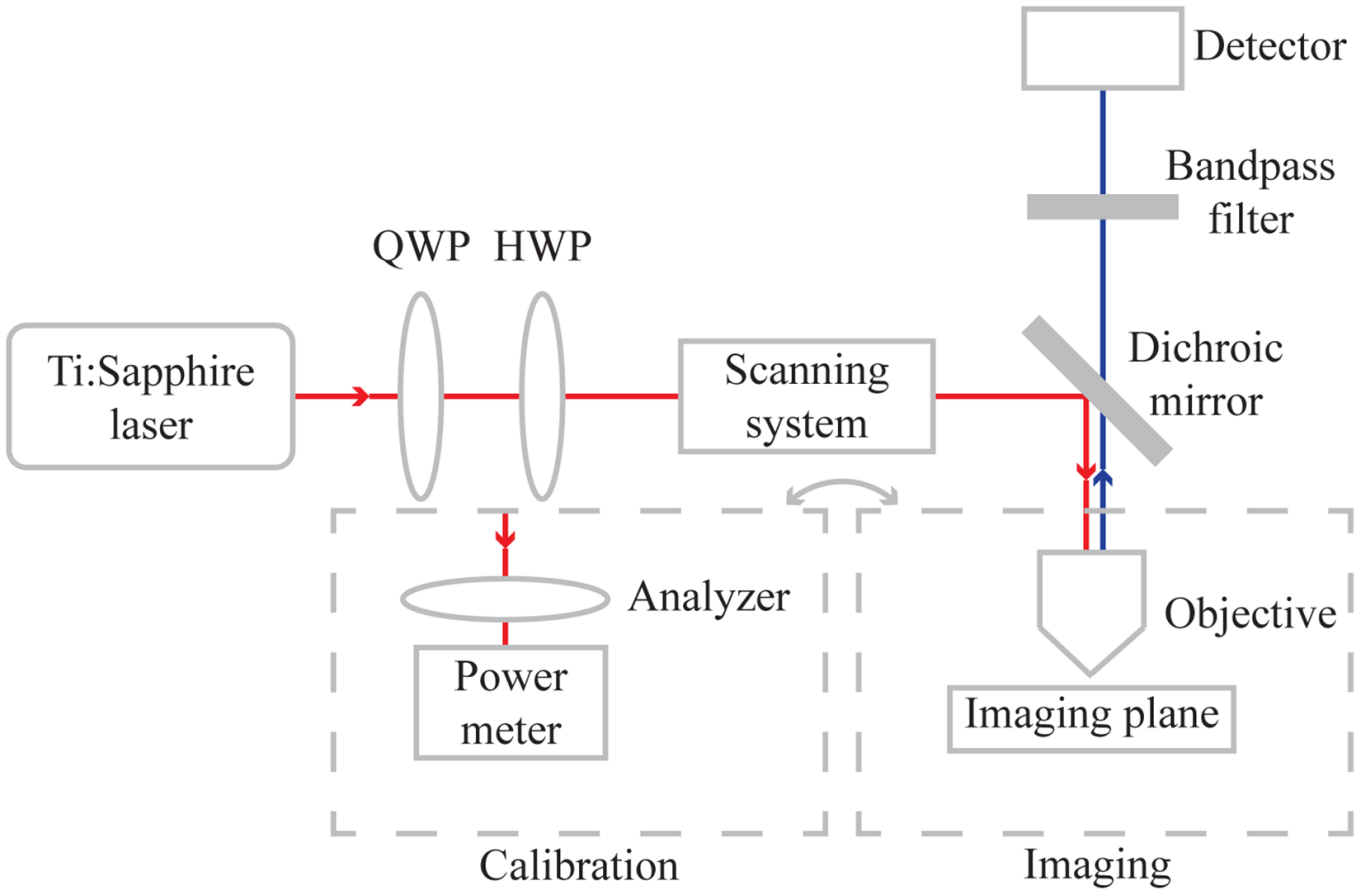
A schematic of the optical setup. A polarization control consisting of a QWP and HWP was mounted between the Ti:Sapphire laser and commercial microscope. The main optical components in the commercial microscope prior to the imaging plane are the scanning system, dichroic mirror and objective. To collect SHG signal a bandpass filter and a HyD detector are employed. To do the calibration of the polarization control the objective is replaced by an analyzer and a power meter.

A polarization control was tested on a Leica TCS SP8 confocal microscope. This microscope was equipped with a Ti:Sapphire laser (Chameleon Vision-S, Coherent), which was tuned to a wavelength of 890 nm for second harmonic generation. The polarization control consisted of a QWP (AQWP10M-980, Thorlabs) and HWP (AHWP10M-980, Thorlabs), mounted in motorized rotation stages (PRM1/MZ8, Thorlabs), placed in the laser beam path before the microscope (see Fig 2). The function of the QWP was to control the ellipticity of the beam, where after the HWP rotated the polarization. In addition, to test the potential for calibrating different microscopes with different optical elements, the calibration procedure was also performed on a Zeiss LSM 510 Meta confocal microscope, equipped with a Ti:Sapphire laser (Mira, Coherent) tuned to a wavelength of 780 nm. An analyzer (LPNIR100, Thorlabs), also mounted in a motorized rotation stage, in combination with a power meter (Labmax-TOP, Coherent) were used to measure the polarization at the imaging plane. The objective was removed when using the analyzer and power meter. Polarization was determined by measuring the laser power as a function of analyzer angle and fitting the measurements to Eq 7. The analyzer was rotated by increments of 30° from 0° to 180°. The sampling rate of the power meter was set to 10 Hz, and the measured intensity at each angle was the average of 10 samples (i.e. 1 s per measurement).

All motors and the power meter were controlled using Matlab (R2015a 32-bit, Mathworks). [18]

### Calibration

To calibrate the polarization setup the polarization at the imaging plane was mapped as a function of QWP and HWP angle. The QWP and HWP were rotated from 0° to 180° and 90°, respectively, at increments of 10°. These measurements were used to fit Eq 3, where four input variables (*I*_*α*_, *ϕ*′, *θ*′ and *α*′) were used to estimate six unknown parameters (*δ*, *γ*, *ϕ*_0_, *θ*_0_, *α*_0_ and $I_{0}=E_{0}^{2}$). The fitting was performed by nonlinear regression using Matlab (function: *nlinfit*). These parameters were used to determine the QWP and HWP angle combinations that provide linearly (Eq 4) and circularly polarized light (Eqs 5 and 6).

On most commercial microscopes it is not possible to simply switch on the laser. During calibration the microscope was therefore continuously scanning. Most scanning microscopes switch off the laser as the scanning mirrors are changing directions and during flyback to protect light sensitive samples. This could affect the measured intensity in an unpredictable way. Measures were taken to reduce the time that the laser was switched off, such as using a single line bidirectional scan. A high scan speed was used to ensure that all intensity measurements were affected relatively equally by the laser switching off when outside the imaging frame (i.e. many scans for every measurement). To reduce the effect of noise on the intensity measurements, maximum laser power was used during the calibration.

To get an impression of the accuracy of the calibration the polarization was measured for a QWP and HWP combination, (*ϕ*′, *θ*′), that should provide linear and circular polarization. The measured QWP and HWP settings were (80°,13°) and (125°,34°), respectively. To assess whether the system was capable of generating a higher degree of linearly and circularly polarized light than provided by the calibration, the polarization was measured at smaller angle increments (2°) for certain QWP and HWP angular ranges ($\phi_{min}^{\prime }-\phi_{max}^{\prime }$, $\theta_{min}^{\prime }-\theta_{max}^{\prime }$). These ranges were (50°-100°, 15°), (50°-100°, 35°) and (119°-131°, 26°-40°), where the first two ranges surround settings that should provide linearly polarized light (80°,15°) and (68°,35°), respectively, and the final range surrounds a setting that should provide circularly polarized light, i.e. (125°,34°). The calibration on the Zeiss microscope naturally provided different QWP and HWP combinations, (*ϕ*′, *θ*′), that should provide linear and circular polarization. Similarly to the other microscope, the polarization was measured round two settings, (90°,6°) and (152°,14°), which should correspond to circularly and linearly polarized light, respectively, to get an impression of accuracy of the calibration.

There are two separate QWP and HWP settings that provide linear polarization with the same orientation. To examine whether there was a difference between the two settings the polarization was measured for both, with settings that should provide linearly polarized light oriented at 0-180° at 10° intervals. The same polarization ranges were also used to image tendon.

Polarization dependent attenuation, *γ*, caused by optical elements in the microscope results in a modulation of the laser power for different angles of linear polarization. For a given linear polarization, corresponding to a specific (*ϕ*′, *θ*′), the expected intensity as a function of analyzer angle *I*_*α*_ can be calculated using Eq 3. The peak intensity of the calculated *I*_*α*_ will vary for different polarizations due to the polarization dependent attenuation *γ*. The peak intensity for a given polarization setting is conveniently determined by fitting *I*_*α*_ to Eq 7 to determine $I_{max}=E_{max}^{2}$.

A certain polarization setting ($\phi_{ref}^{\prime }$, $\theta_{ref}^{\prime }$) was chosen as reference and a power calibration factor *r* was calculated using

$$\begin{array}{c} r\left(\phi^{\prime },\theta^{\prime }\right)=\frac{E^{2}{\left(\phi^{\prime },\theta^{\prime }\right)}_{max}}{E^{2}{\left(\phi_{ref}^{\prime },\theta_{ref}^{\prime }\right)}_{max}} \end{array}$$ (8)

This factor could be used to adjust the excitation power during imaging. However, due to limitations in the software and APIs of many microscopes it can be difficult to adjust the laser power for every image. We therefore opted to adjust the signal post imaging. This choice can be changed on other platforms. The acquired image was then calibrated using

$$\begin{array}{c} I_{SHG}^{\prime }=rI_{SHG}. \end{array}$$ (9)

Here *I*_*SHG*_ is the original intensity of the image and *r* is the estimated ratio between the laser power (that actually reaches the sample) used to image the reference image and this image.

### Uncertainty prediction

The QWP and HWP settings (*ϕ*′ and *θ*′) that correspond to circular and linear polarization were determined by Eqs 5 and 6 for circular and Eq 4 for linear polarization. However, the parameters that enter into these equations have an inherent uncertainty which was quantified by the covariance matrix returned by the fitting of Eq 3.

For circular polarization, Eqs 5 and 6 can be equated and solved to provide a function for *ϕ*′(*ϕ*_0_, *δ*, *γ*), and either of these functions can be solved to yield a function for *θ*′(*ϕ*′, *θ*_0_, *ϕ*_0_, *δ*, *γ*). In the latter, *ϕ*′ was assumed to be constant with no uncertainty when determining the uncertainty of *θ*′. Propagation of error was then used to calculate the uncertainty in *ϕ*′ and *θ*′ based on the uncertainty given by the covariance matrix of the arguments.

For linear polarization, there is a line in the (*ϕ*′, *θ*′) plane that corresponds to different direction of the linear polarization. We therefore introduce a new variable *x* = 2*θ*′ − *ϕ*′, where *x* ∈ [0°, 180°]. We can then use Eq 4 to write down a function for *ϕ*′(*x*, *θ*_0_, *ϕ*_0_, *δ*, *γ*). The variable *x* is assumed to have no uncertainty in this expression but is varied in the range *x* ∈ [0°, 180°]. Using propagation of error for this expression provides an uncertainty interval around the curve *ϕ*′(*x*). This can then be converted to an uncertainty interval in the (*ϕ*′, *θ*′) plane by the expression for *x*.

### Sample imaging

Tendon was used as a model tissue to test the polarization calibration. The sample was excised from a chicken specimen obtained commercially and post-mortem. Tendon was colored by erythrosine (to aid in locating sample during sectioning), placed in Tissue-Tek and the whole sample was frozen using liquid nitrogen. The sample was sliced at a thickness of 50 μm along the length of the tendon. Before sealing the slide with a coverslip the sample was unfrozen and 70% glycerol was added. The sample was thereafter stored at 4°C.

After calibrating the polarization setup, the power meter and analyzer were removed and a 10×, 0.40 NA objective was added. All other filters and optics were the same. In the epi-direction a HyD detector combined with a 435-455 nm bandpass filter was used to record the backward scattered SHG. Between each image frame the linearly polarized laser light was rotated in increments of 10°, until a range of 180° was covered, resulting in a total of 19 images. All images were 512 × 512 pixels and covered an area of 1.16 × 1.16 mm. Two different combinations of QWP and HWP angles provide the same linearly polarized light. To test if there was a difference between these two setting, both combinations were used for imaging, resulting in two sets containing 19 images each. To have an indication of reproducibility both sets were collected twice.

Using linear least squares fitting as described in detail by Rouède et. al. [19] the second-order susceptibility tensor ratios, χ_33_/χ_31_ and χ_15_/χ_31_, and in plane fiber angle, *φ*, were determined based on the polarization measurements. Prior to analysis a 3 × 3 averaging filter was applied and all pixels that were saturated or had a low intensity (average gray value lower than 10) were excluded.

## Results

**Fig 3:**
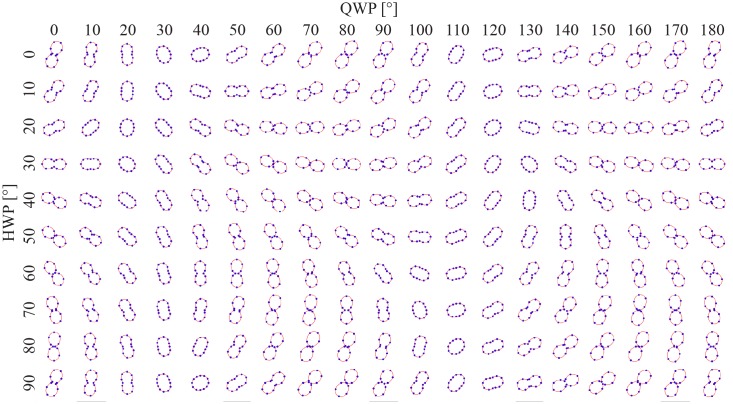
The laser polarization at the imaging plane measured as a function of the QWP and HWP angle. The blue dots represent measurements at different analyzer angles, while the red curves were determined by fitting Eq 7. A circular curve corresponds to circular polarization, a figure-eight shaped curve corresponds to linear polarization. The laser is elliptically polarized if the shape is somewhere in between.

An illustration of the data collected during a calibration is provided in Fig 3. Here, the polarization of the laser at the imaging plane is illustrated as a polar plot and mapped as a function of the QWP and HWP angle. The measured intensity at different analyzer angles are shown as blue dots. For every QWP and HWP setting, these measurements are fitted to Eq 7. The resulting graph from this fitting is the superimposed red curve. When the polarization is circular the intensity curve is circular, if the polarization is linear the intensity curve has a figure-eight shape. The fitting also provides the parameters *E*_*min*_ and *E*_*max*_ which are used to provide a numerical value for the ellipticity of the polarization

$$\begin{array}{c} \rho =\frac{E_{min}}{E_{max}}. \end{array}$$ (10)

**Fig 4:**
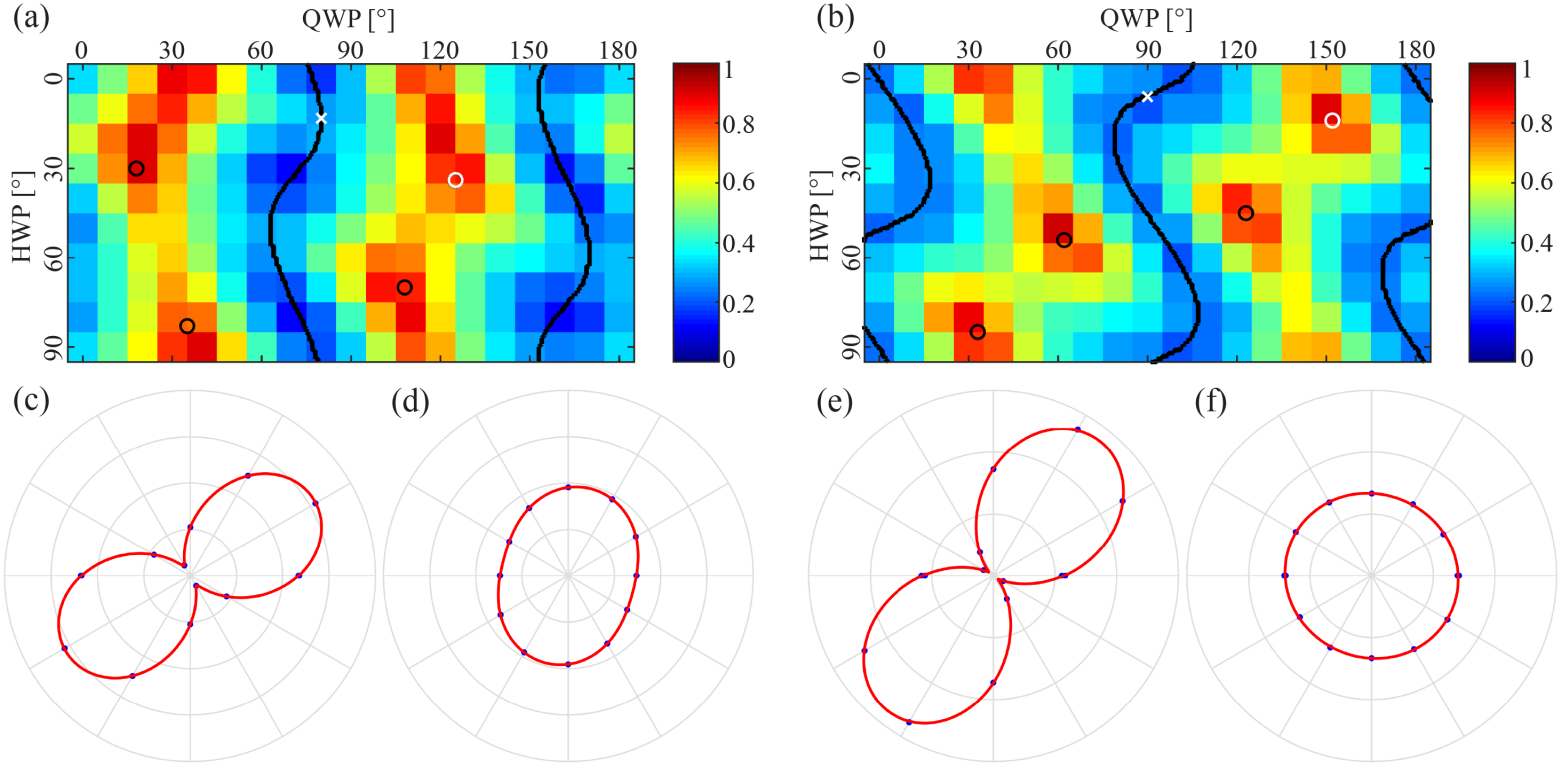
(a, b) An overview of the ellipticity, *ρ*, between the minimum and maximum electric field component of the polarization for different HWP and QWP combinations for two different microscopes. For linearly polarized light the ellipticity is 0, and for circularly polarized light the ellipticity is 1. The overlaying plots indicate which waveplate combinations generate linearly polarized light (black lines) and circularly polarized light (black and white circles) as determined by fitting a theoretical model. The white crosses in each plot indicates the HWP and QWP settings used when measuring the polarization illustrated in (c) and (e). The white circles correspond similarly to plots (d) and (f). The single measurements had an ellipticity of *ρ* = 0.29 (c), *ρ* = 0.86 (d), *ρ* = 0.19 (e) and *ρ* = 0.97 (f).

An overview of the ellipticity as a function of QWP and HWP angles is given in Fig 4a. To be able to set the polarization state, the unknown parameters in the model (see the calibration section) were determined by fitting the measurements to Eq 3. Eqs 4–6 where then used to find the settings of the QWP and HWP that correspond to linear and circular polarization. These settings are shown as lines and circles in Fig 4 for linear and circular polarization respectively. It can be seen that the linear polarization (lines) corresponds to regions of low ellipticity and circular polarization (circles) corresponds to regions of high ellipticity.

The calibration was also performed on an additional microscope (Zeiss LSM 510 Meta) and the results are shown in Fig 4b. The optics in this microscope introduced a different phase and attenuation on the polarization of the laser, creating a different correspondence between the QWP and HWP angle and the polarization measured at the imaging plane. However, for both cases the QWP and HWP combinations determined to provide linearly and circularly polarized light visually match the map of polarization measurements.

Measuring the polarization at a QWP and HWP setting that should correspond to circularly and linearly polarized light demonstrates that the calibration seems to be slightly better for the second microscope, where *ρ* = 0.19 for linearly polarized light (Fig 4e) and *ρ* = 0.97 for circularly polarized light (Fig 4f), compared to the first microscope, where *ρ* = 0.29 for linearly polarized light (Fig 4c) and *ρ* = 0.86 for circularly polarized light (Fig 4d).

**Fig 5:**
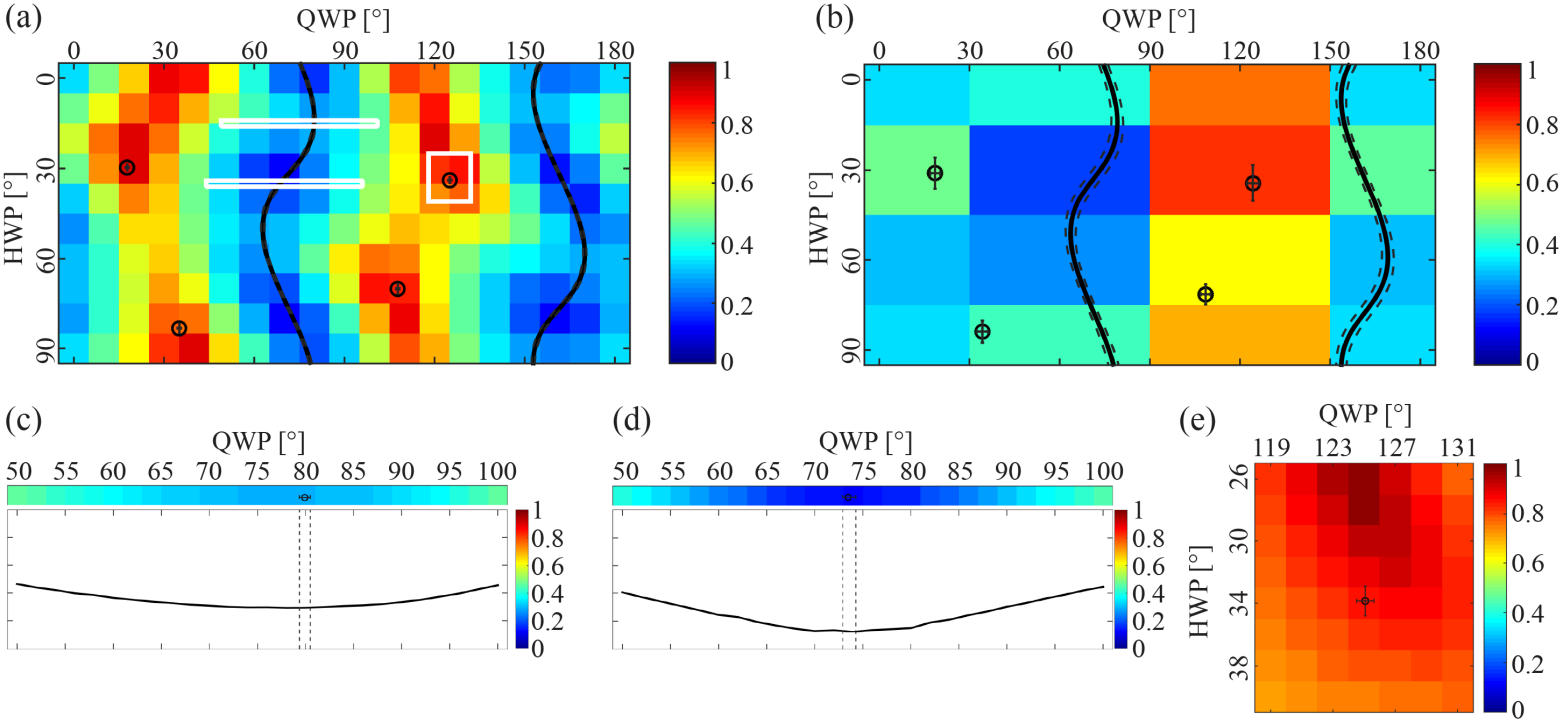
(a, b) A detailed and coarser map of the ellipticity, *ρ*, with overlaying waveplate combinations that provide linearly (mean: black lines, ±3std: dashed lines) and circularly (mean: black circles, ±3std: intervals) polarized light. To illustrate the accuracy smaller regions of interest (a, white rectangles) were measured in more detail (c, d, e). The overlapping plots are based on the fitting of the original map (a), and have an ellipticity of *ρ* = 0.29 (c), *ρ* = 0.12 (d) and *ρ* = 0.86 (e).

By illustrating the uncertainty in the determined QWP and HWP settings the accuracy of the calibration can be visualized. The calculated uncertainty in the determined QWP and HWP angles are illustrated as error bars and dashed lines in Fig 5a for the circular and linear polarization respectively. It can be seen that the uncertainty is very small and almost indistinguishable.

Reducing the number of measurements that are used for fitting the data (see Fig 5b). does not seem to alter the resulting fit to a large degree except for increasing the standard deviations.

Fig 5c and 5d show details of the measured polarization in the white strips in Fig 5a. It can be seen that the calibrated waveplate settings is close to the ideal settings (i.e. lowest ellipticity) that provide the most linearly polarized light. However, the minimum ellipticity is slightly different (*ρ* = 0.29 and 0.12) for the two regions.

Fig 5e shows details of the polarization data in the white rectangle in Fig 5a. It can be seen that the settings for circularly polarized light is slightly off mark with a HWP angle of approximately 6°, and a resulting ellipticity of *ρ* = 0.86.

**Fig 6:**
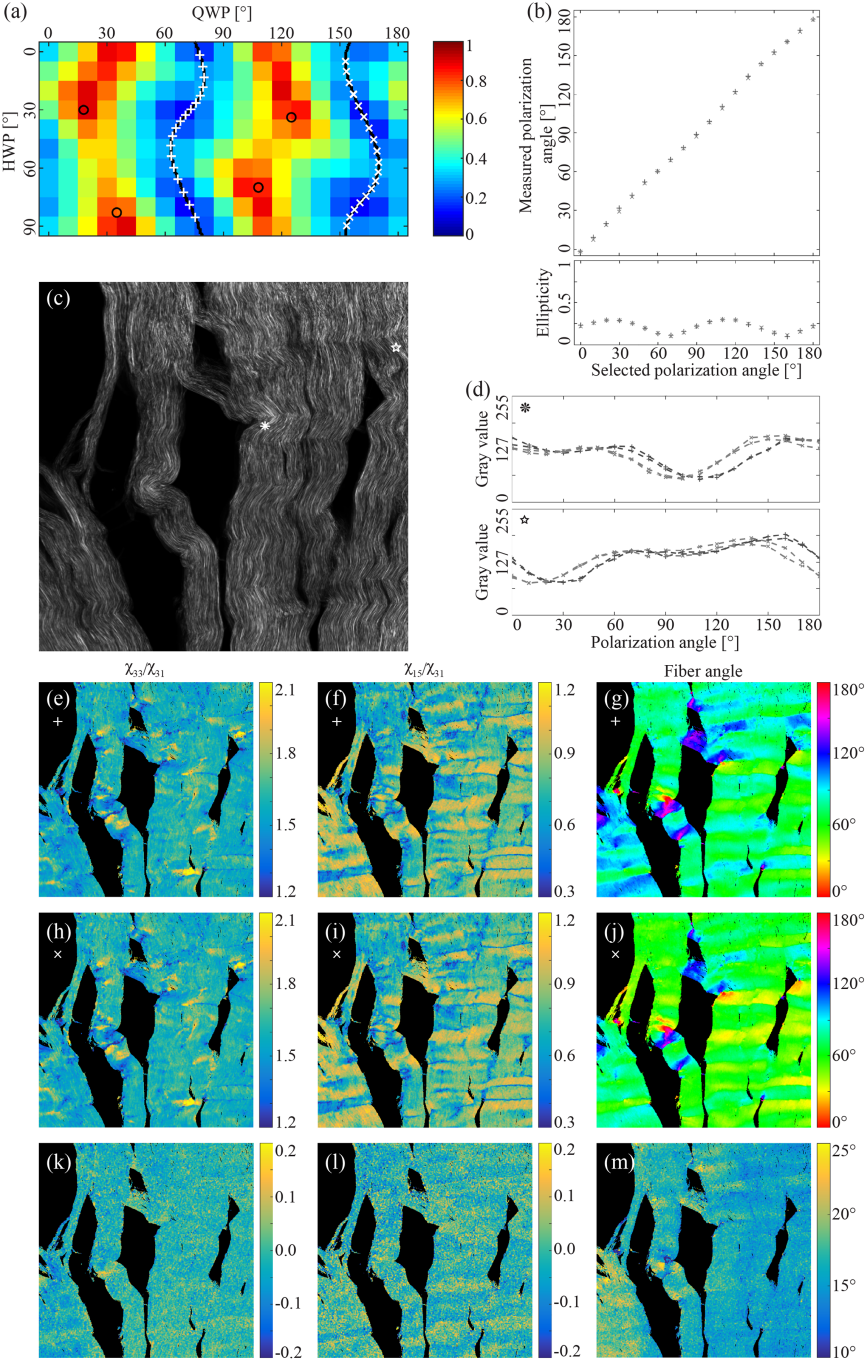
Waveplate combinations that provide linear polarization were selected at polarization angle intervals of 10° from 0° to 180° (a, white crosses and plus signs). There are two different combinations that provide the same polarization angle, thereof the two separate sets (crosses vs plus signs). The polarization angle and ellipticity were measured for all waveplate combinations in both sets (b). Tendon was imaged twice using all waveplate combinations (c, average). Two pixels were selected (c, white asterisk and star) to demonstrate the variation of the intensity as a function of polarization angle for the two sets (d). The second order susceptibility tensor ratios and fiber angle were calculated separately for the two sets (plus signs: e, f, g, crosses: h, i, j). The difference between the two was emphasized by taking subtracting the results from one of the sets (crosses) from the other (plus signs) (k, l, m).

There are two different waveplate settings that provide linearly polarized light of a certain polarization angle. This is illustrated as the ‘+’ and ‘x’ in Fig 6a. Fig 6b shows the measured polarization angle and the ellipticity for these two settings. The results show that the settings result in basically identical polarization states.

Fig 6d shows the polarization dependence of the two points marked in Fig 6c for the two different settings of achieving a polarization state. Even though the results in Fig 6b indicates that the polarization states are identical, the resulting polarization dependence of the sample is somewhat shifted. This is not due to a measurement error or systematic noise, since repeating the measurements gave minimal changes.

Fig 6e–6j show the calculated susceptibility ratios as well as the calculated fiber angle for the two different settings. Fig 6k and 6l show that difference of the two images above in the same column. The polarization measurements indicate that the susceptibility ratios is similar, but the determined fiber angle is shifted by approximately 15° when comparing the two settings.

## Discussion

The main purpose of this study was to develop an automated and user-friendly system for calibration and control of the polarization of the laser on a commercial microscope for P-SHG. Manual operation and calibration is time consuming and prone to errors. This system thus opens up to possibility for higher throughput and more accurate results.

The automated system conveniently provides an overview of the relation between QWP and HWP angle and the polarization at the imaging plane (Fig 3). For an ideal system, with no influence from optics in the microscope, the QWP determines the ellipticity, and the HWP rotates the polarization. This overview directly illustrates that the optics in the microscope prior to the objective influences the polarization of the laser, since the lasers ellipticity is not independent of the HWP angle, demonstrating the need to precompensate for this alteration. Fig 4a and 4b illustrates that the effect and resulting calibration varies from system to system.

In theory, a HWP should be sufficient to determine the phase and attenuation caused by the optics in the microscope, as introduced by Chou et. al. [15]. However, in our experience the data provided by rotating only the HWP is not enough to estimate the correct QWP and HWP combinations for linear and circular polarization. Measuring the polarization as a function of both the QWP and HWP angle as part of the calibration is more likely to provide an accurate fitting, also enabling visual verification by plotting the results onto the ellipticity map, as illustrated in Fig 4a and 4b.

Theoretically the QWP and HWP should be able to correct for the relative phase shift introduced by the microscope. However, there is a difference in the achieved linearity of the polarization depending on the WP settings (Fig 5c and 5d). The difference in ellipticity (*E*_*min*_/*E*_*max*_), *ρ* = 0.29 and 0.12, is not caused by attenuation, since *E*_*max*_ is approximately the same for both (values not included). The four areas with higher linearity (dark blue areas in Fig 5a) correspond to the WP combinations that provide linearly polarized light parallel and perpendicular to the optical axis along which a phase is introduced by the optics in the microscope. This was deduced by examining the resulting fitting variables (*ϕ*_0_ = 71° and *θ*_0_ = 32°), also the difference in QWP and/or HWP angle of these areas are 90° and 45°, respectively. This shows that the phase introduced by the optics in the microscope is not completely compensated for by the QWP and HWP. A reason could be that the QWP and/or HWP are not behaving as they ought to, which could be caused by inaccurate alignment of these filters compared to the laser beam path. The polarization map of the second microscope, Fig 4b, does not seem to display this variation in ellipticity (since the ellipticity varies little along the QWP and HWP coordinates that provide linearly polarized light). This indicates that an equally degree of linear polarization at different polarization angles might not be achieved on every system, and if this is desired the calibration has to be controlled and might be improved by realigning the QWP and/or HWP.

The standard deviation interval deduced from the fitting does not contain the QWP and HWP angular combination that provides exact circular polarization (Fig 5e). This indicates that there is a systematic error, which could for example be caused by inaccurate alignment of the QWP and HWP compared to the laser beam path. The exact QWP and HWP combination that provides circular polarized light can be obtained from more detailed mapping of the polarization as a function QWP and HWP angle, similar to the measurements displayed in Fig 5e.

A detailed ellipticity map, such as in Fig 5a, takes about 2 hours to measure. In comparison, a coarser map, as in Fig 5b, provides approximately the same fitting results, and takes about 10 minutes to run. Optimization of the Matlab code used to run the calibration can further reduce the time required, an example being arranging the calibration sequence such that the motors are rotated as little as possible and simultaneously, since the mechanical rotation is currently the most time-consuming process during the calibration. Any change of filter or dichroic mirror will change the phase and reflection introduced in the microscope and the calibration has to be reapplied, indicating the need for an automated calibration process on a commercial microscope.

There are two WP combinations that generate linearly polarized light with the same polarization angle at the imaging plane (Fig 6b). However, the SHG signal generated by tendon is slightly shifted according to the polarization angle depending on which WP combination is selected (Fig 6d). This is reflected in the calculation of the second order susceptibility tensor ratio. These are similar for both settings, while the determined fiber angle is slightly different (Fig 6e–6m). This is not of grave consequence because the determined fiber angle is a relative angle, and its oriented in the imaging plane has to be estimated by visual inspection. However, mixing the two separate sets of polarization settings might influence the calculation of the tensor ratios.

## Conclusion

In this study a setup to automatically calibrate and control the polarization of the laser on a commercial microscope has been proposed. The automation decreases the time required to calibrate the polarization setup compared to manual calibration. As demonstrated this makes it possible to analyze and retrieve information about both the results from the calibration and its relation to P-SHG that would not have been possible with a manually operated system.
